# Aneuploidy generates enhanced nucleotide dependency and sensitivity to metabolic perturbation

**DOI:** 10.1101/gad.352512.124

**Published:** 2025-06-01

**Authors:** Rayna Y. Magesh, Arshia N. Kaur, Faith N. Keller, Abdulrazak Frederick, Tenzin Tseyang, John A. Haley, Alejandra M. Rivera-Nieves, Anthony C. Liang, David A. Guertin, Jessica B. Spinelli, Stephen J. Elledge, Emma V. Watson

**Affiliations:** 1 Department of Systems Biology, University of Massachusetts Chan Medical School, Worcester, Massachusetts 01605, USA;; 2 Program in Molecular Medicine, University of Massachusetts Chan Medical School, Worcester, Massachusetts 01605, USA;; 3 Diabetes Center of Excellence, University of Massachusetts Chan Medical School, Worcester, Massachusetts 01605, USA;; 4 Department of Genetics, Harvard Medical School, Boston, Massachusetts 02115, USA;; 5 Division of Genetics, Department of Medicine, Brigham and Women's Hospital, Boston, Massachusetts 02115, USA;; 6 Howard Hughes Medical Institute, Chevy Chase, Maryland 20815, USA

**Keywords:** CRISPR, aneuploidy, cancer, genomics, metabolism

## Abstract

Despite the general detriment of aneuploidy to cellular fitness, >90% of solid tumors carry an imbalanced karyotype. This existing paradox and the molecular responses to aneuploidy remain poorly understood. Here, we explore these cellular stresses and unique vulnerabilities of aneuploidy in human mammary epithelial cells (HMECs) enriched for breast cancer-associated copy number alterations (CNAs). To uncover the genetic dependencies specific to aneuploid cells, we conducted a comprehensive, genome-wide CRISPR knockout screen in isogenic aneuploid and diploid HMEC lines. Our study reveals that aneuploid HMECs exhibit an increased reliance on pyrimidine biosynthesis and mitochondrial oxidative phosphorylation genes and demonstrate heightened fitness advantages upon loss of tumor suppressor genes. Using an integrative multiomic analysis, we confirmed nucleotide pool insufficiency as a key contributor to widespread cellular dysfunction in aneuploid HMECs with net copy number gain. Although diploid cells can switch seamlessly between pyrimidine synthesis and salvage, cells with increased chromosomal content exhibit p53 activation and S-phase arrest when relying on salvage alone, alongside increased sensitivity to DNA-damaging chemotherapeutics. This work advances our understanding of the consequences of aneuploidy and uncovers potential avenues for patient stratification and therapeutic intervention based on tumor ploidy.

## Abstract (precis)

In this study, Magesh et al. describe the metabolic consequences of aneuploidy on mammary epithelial cells with breast cancer-associated copy number alterations. They identify metabolic dependencies, particularly in nucleotide biosynthesis and salvage, as well as mitochondrial oxidative phosphorylation and glycolysis, of net gain aneuploid cells and uncover their implications for improving chemotherapeutic response.

Aneuploidy, the state of having abnormal numbers of chromosomes, contributes to numerous human disorders, including Down syndrome, infertility/miscarriage, mosaic variegated aneuploidy syndromes, sex chromosomal abnormalities, and cancer. Notably, tumors often exhibit exceptionally high levels of somatic aneuploidy, affecting on average 20%–30% of the genome (Beroukhim et al. 2010; Watson et al. 2024). Aneuploidy is considered a hallmark of cancer (Weaver and Cleveland 2006) and is observed in 90%–95% of solid cancers. Most tumor genomes evolve within a 2N to 4N ploidy range (Cheng et al. 2017) (30%–40% undergoing whole-genome doubling) and display tissue-specific karyotype selection patterns (Beroukhim et al. 2010). Aneuploidy has been proposed to provide proliferative or survival benefit during tumorigenesis (Guscott and McClelland 2024; Watson et al. 2024) by shaping tumor suppressor and oncogene dosage (Davoli et al. 2013; Sack et al. 2018). However, aneuploidy also induces cellular stress and is generally detrimental to cellular fitness (Torres et al. 2007; Williams et al. 2008; Oromendia et al. 2012; Sheltzer et al. 2017; Zhu et al. 2018). How tumor cells overcome the burdens associated with an imbalanced genome, often a net excess of chromosomes, is not well understood but may represent exploitable nodes of vulnerability in cancer (Cohen-Sharir et al. 2021; Marquis et al. 2021; Quinton et al. 2021; Payton et al. 2024).

The burden of excess chromosomes in net gain aneuploidy induces stress across a myriad of biochemical systems that synthesize the polymers of life: DNA, RNA, and protein. Just how widespread these stresses permeate across cellular systems is still under investigation, with documentation of significant disruption to replication (Burrell et al. 2013; Passerini et al. 2016; Wangsa et al. 2018), transcription (Ben-David et al. 2014; Torres et al. 2016; Hwang et al. 2021; Watson et al. 2024), RNA metabolism (Ippolito et al. 2024), autophagy (Stingele et al. 2012; Liu et al. 2016), and proteostasis (Oromendia et al. 2012; Ohashi et al. 2015; Chunduri and Storchová 2019). In yeast, *Drosophila*, and mouse embryonic fibroblasts, chromosomal imbalance results in metabolic disruptions in the form of increased ROS production (Li et al. 2010; Dephoure et al. 2014; Shaukat et al. 2014). It is no surprise, then, that aneuploidy generally slows growth rates in experimental models ranging from yeast to human cell lines, with the exception of some recurrent copy number alterations found prevalently in cancer, which have been shown to increase growth rate in cognate “tissue of origin” models (Watson et al. 2024).

Net gain aneuploidy is associated with p53 activation and apoptosis in isogenic epithelial model systems (Li et al. 2010; Ohashi et al. 2015) and systemically in Down syndrome (Anderson et al. 2000; Tramutola et al. 2016), which has been proposed to derive from aneuploidy-associated replication stress (Wangsa et al. 2018). This likely goes beyond gene dosage effects, because cells from patients with sex chromosomal abnormalities caused by an extra copy of the inactive X chromosome (Klinefelter syndrome [XXY] or triple X syndrome [XXX]) also exhibit slowed cell cycles (Barlow 1972, 1973a,b; Otter et al. 2010) and altered expression of cell cycle and metabolic genes (Blanton et al. 2024; San Roman et al. 2024). Aneuploidy-associated replication stress and p53 activation can lead to growth arrest, senescence, and/or apoptosis (Andriani et al. 2016; Santaguida et al. 2017; Watson and Elledge 2017). Thus, one function of p53 is to sense and restrict aneuploidy in the body, and loss of *TP53* by somatic mutation in tumors is strongly associated with increased levels of aneuploidy compared with tumors without *TP53* mutations (Ciriello et al. 2013; Taylor et al. 2018; Redman-Rivera et al. 2021). In fact, patients with Li-Fraumeni syndrome who harbor germline *TP53* mutations have increased levels of aneuploidy in tissues and develop many different types of cancer, often within the same patient (Shlien et al. 2008). p53 is the top growth-restricting factor in aneuploid RPE1 cells, as determined through unbiased functional genomic methods (Ippolito et al. 2024; Zerbib et al. 2024). However, the underlying mechanisms by which aneuploidy leads to replication stress and subsequent p53 activation are poorly understood.

Here we used a functional genomics approach to characterize gene essentiality in net gain aneuploid human mammary epithelial cells compared with isogenic diploid controls. By performing genome-wide CRISPR screens (Wang et al. 2014; Doench et al. 2016) in paired aneuploid/diploid mammary cell lines (Watson et al. 2024) and integrating transcriptomic, metabolomic, and bioenergetics data, we identified nucleotide pool insufficiency as a key underlying mechanism of p53 activation in net copy number gain aneuploid cells. Additionally, aneuploid cells exhibited increased mitochondrial ATP production and glycolytic rates to support the heightened metabolic demands associated with supernumerary chromosomal content. Nucleotide pool insufficiency associated with net gain chromosomal aneuploidy could potentially be targeted to enhance current chemotherapeutic modalities of breast cancer treatment or stratify patients under current clinical standards of care.

## Results

### Genome-wide aneuploidy synthetic lethality CRISPR screens

**Figure 1.**
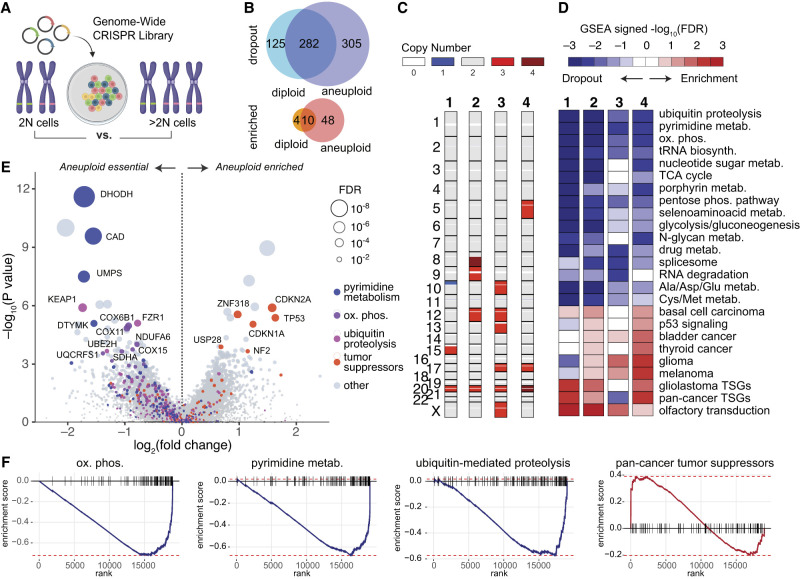
Net gain aneuploidy epistasis profile in human mammary epithelial cells. (*A*) Diagram of concept for genome-wide aneuploidy CRISPR screens. Four unique aneuploid HMEC clones with mostly nonoverlapping CNAs were used for the screen alongside a paired, isogenic diploid control. (*B*) Diagram showing the shared and unique differentially enriched/depleted genes between isogenic aneuploid and diploid HMECs. (*C*) Bar columns indicate the copy number profiles of each of the four unique aneuploid clones. (*D*) The top enriched/depleted pathways across each of the four aneuploid clone screens, each compared with their isogenic diploid control. (*E*) Volcano plot showing gene-level meta-analysis of all aneuploid versus diploid screens, with specific genes colored based on their pathway involvement in pyrimidine metabolism, oxidative phosphorylation, and ubiquitin-mediated proteolysis. Enriched tumor suppressor genes are also labeled. (*F*) Enrichment plots for oxidative phosphorylation genes, pyrimidine biosynthesis genes, ubiquitin-mediated proteolysis genes, and pan-cancer tumor suppressor genes in aneuploid HMEC screens compared with diploid control screens.

With the goal of fully profiling aneuploidy synthetic lethalities in a cancer-relevant system, we performed genome-wide CRISPR screens in isogenic diploid and aneuploid cell lines derived from diploid hTERT immortalized human mammary epithelial cells (hTERT HMECs, referred to here as “HMECs”), as previously described and validated by Watson et al. (2024). HMECs are considered good models for the cell type of origin of multiple breast cancer subtypes (Herbert et al. 2002; Zhao et al. 2003). Additionally, HMECs are untransformed and do not contain driver mutations as assessed by deep whole-genome sequencing (Watson et al. 2024), enabling us to isolate the aneuploidy synthetic lethality profile without the complications of background mutational status. We chose four different aneuploid clonal lines with largely nonoverlapping chromosomal amplifications, except for chromosome 20 gain, which was recurrently selected in HMECs in vitro (Watson et al. 2024) and was present in each line. We also performed control screens using three different clonally derived diploid lines in duplicate for a total of 14 genome-wide CRISPR screens (Fig. 1A). This library contained five guides per gene targeting ∼18,000 protein-coding genes for a total of ∼90,000 reagents (Supplemental Table S1).

After infection with the lentiviral CRISPR library at a multiplicity of infection of 0.3, the initial “population doubling 0” (PD0) time point was collected with representation of 500 cells per guide. Cells were then passaged for six population doublings and collected for the end point of the screen (PD6). Gene dropout/enrichment was determined based on sequencing coverage of guides after amplicon-seq of the extracted genomic DNA using edgeR (Robinson et al. 2010; Ritchie et al. 2014). A slight bias in dropout/enrichment based on chromosomal location for genes on chromosomes affected by aneuploidy was observed as described previously (Meyers et al. 2017), which we resolved by implementing a computational correction (Supplemental Fig. S1A).

A start-to-end point analysis of aneuploid and diploid screens revealed largely overlapping gene dropout and enrichment profiles; however, more genes were essential in aneuploid HMECs compared with diploid HMECs (cutoff FDR <0.05 and log_2_ fold change <−2) (Fig. 1B; Supplemental Fig. S1B; Supplemental Tables S2, S3). To generate aneuploidy epistasis profiles, we compared PD6 end points between the aneuploid and diploid screens (Fig. 1C–F; Supplemental Tables S4, S5). Gene set enrichment analysis of epistasis profiles for each of the four aneuploid mutants used in the screens revealed differential dropout of guides targeting genes in pathways known to be stressed in aneuploidy, such as ubiquitin-mediated proteolysis (Fig. 1D–F). Additionally, we observed significantly stronger enrichment of p53 pathway targeting guides (as well as other tumor suppressor pathways) in aneuploid lines compared with diploids (Fig. 1D–F; Supplemental Fig. S1B,C). The top 25 aneuploidy-enriched hits in three out of four screens included the prominent tumor suppressors TP53, CDKN1A, USP28, NF2, and CDKN2A (Fig. 1E; Supplemental Fig. S1C).

### Net gain aneuploid cells do not tolerate de novo pyrimidine synthesis disruption and are insufficiently rescued with uridine

**Figure 2.**
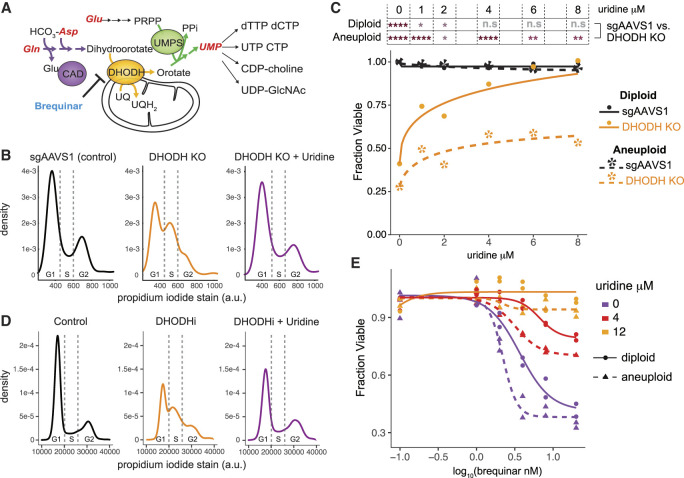
Net gain aneuploid cells are sensitive to pyrimidine synthesis disruption. (*A*) Diagram of the pyrimidine biosynthesis pathway. (*B*) Density plots showing DNA stained with propidium iodide in parental HMECs with *AAVS1* (control) knockout, *DHODH* knockout, and *DHODH* knockout supplemented with uridine. *DHODH* knockout causes cells to arrest in S phase, which is rescued by uridine. (*C*) Cell viability of *AAVS1* and *DHODH* knockout HMECs in diploid and net gain aneuploid (clone 2 from Fig. 1C) backgrounds under uridine supplementation ranging from 0 to 8 μM. The significance of the difference between the *AAVS1* knockout and the *DHODH* knockout cell viability in aneuploid and diploid backgrounds was calculated with *t*-tests at each uridine dose assayed and is indicated by the asterisks in the table *above* the graphs. (****) *P*-value < 0.0001, (***) *P*-value < 0.001, (**) *P*-value < 0.01, (*) *P*-value < 0.05. The aneuploid HMEC line used for this experiment was clone 2 in Figure 1C. (*D*) The DHODH inhibitor (DHODHi) brequinar phenocopies *DHODH* knockout. (*E*) Cell viability of diploid (solid lines/circles) and aneuploid (dotted lines/triangles; clone 1 from Fig. 1C) HMECs after treatment with 0.1–40 nM brequinar doses and 0, 4, and 12 μM uridine doses (depicted using purple, red, and yellow, respectively).

The top differential dropout hits in the aneuploidy screens centered around nucleotide biosynthesis and mitochondrial oxidative phosphorylation metabolic pathways (Fig. 1D–F). The three enzymes that catalyze the first six reactions of the de novo pyrimidine biosynthesis pathway (CAD, DHODH, and UMPS) were among the top four dropout hits (Fig. 1E; Supplemental Fig. S1D). DHODH is positioned in the mitochondrial membrane and catalyzes the vital pyrimidine biosynthesis reaction of dihydroorotate (DHO) reduction to orotate, passing reducing equivalents directly to the mitochondrial ubiquinone pool (Fig. 2A; Boukalova et al. 2020; Spinelli et al. 2021). Thus, nucleotide metabolism and mitochondrial redox biology are inextricably linked, and this critical node renders mitochondrial oxidative phosphorylation indispensable for tumor cell proliferation despite high flux and ATP production through glycolysis (Bajzikova et al. 2019; Martínez-Reyes et al. 2020). Our data suggest that this pyrimidine biosynthesis/mitochondrial oxidative phosphorylation axis is especially critical for proliferation of cells with supernumerary chromosomal content, perhaps due to increased nucleotide and energy requirements.

To validate this dependency, we used CRISPR to mutate *DHODH* in representative aneuploid and diploid mammary epithelial cell lines. During the process of selection and recovery, we supplemented cells with uridine to prevent cell cycle arrest/death. Complete protein loss in each line was confirmed via Western blot (Supplemental Fig. S2A). *DHODH* knockout resulted in S-phase accumulation when uridine was withdrawn from culture in both diploid and aneuploid lines (Fig. 2B; Supplemental Fig. S2B). Although the cell cycle arrest caused by *DHODH* mutation in diploid cells could be fully rescued by supplementing physiological plasma uridine concentrations (3–8 μM) (Zhang et al. 2020), *DHODH* knockout in aneuploid cells could not be fully rescued by the same dose range of uridine supplementation (Fig. 2C). This suggests that net gain aneuploid cells require more pyrimidine than diploid cells to replicate a higher chromosome load. This also indicates that, although diploid mammary epithelial cells are capable of fully sustaining growth using either nucleotide synthesis or nucleotide salvage alone, net gain aneuploid cells cannot proliferate at full capacity using only pyrimidine salvage. This phenomenon could also be recapitulated with the DHODH inhibitor (DHODHi) brequinar (Peters et al. 1987), which at high dose results in S-phase accumulation (Fig. 2D) and differentially affects diploid and aneuploid cell growth under various uridine supplementation conditions (Fig. 2E; Supplemental Fig. S2C). Aneuploid cells entering S-phase arrest induced by DHODHi were less likely than diploid cells to successfully re-enter and progress through the cell cycle when provided uridine after arrest (Supplemental Fig. S2D–F).

### Net gain aneuploid cells display insufficient pyrimidine synthesis relative to ploidy

**Figure 3.**
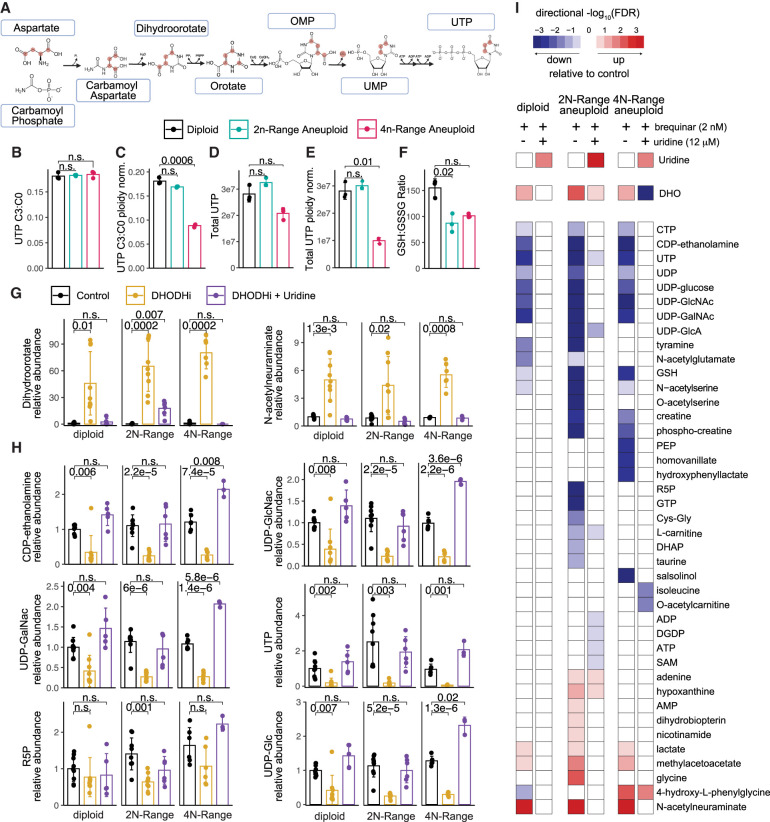
Pyrimidine synthesis inhibition exacerbates nucleotide insufficiency in net gain aneuploid cells. (*A*) Diagram depicting the labeled carbon aspartate tracing through the pyrimidine biosynthesis pathway. (*B*) Bar graphs showing the ratio of labeled UTP to unlabeled UTP from the aspartate tracing experiment in representative diploid, 2N-range aneuploid (clone 2), and 4N-range aneuploid (+1q +2p −4q +7 ++8q −10p +10q +12 +20q −22 −X) HMECs. (*C*) Ratio of labeled UTP to unlabeled UTP normalized for total chromosomal content relative to the diploid (46 chromosome) baseline. (*D*) Total UTP (labeled + unlabeled) levels in each cell type, per-cell-normalized. (*E*) Total UTP (labeled + unlabeled) levels in each cell type, normalized for total chromosomal content. (*F*) Ratio of reduced (GSH) to oxidized (GSSG) glutathione in each cell type. (*G*,*H*) Bar graphs showing the relative abundance of metabolites that increase significantly with low-dose DHODH inhibition (2 nM brequinar), including the DHODH substrate dihydroorotate (*G*), or decrease with DHODH inhibition (*H*) in diploid, 2N-range aneuploid, and 4N-range aneuploid HMECs. *P*-values were calculated using *t*-tests corrected for multiple testing. Data are summarized from biological replicates across multiple representative clones for each ploidy class, described in more detail for *I*. (*I*) Heat map summary of LC/MS-MS-derived metabolomic profiles of diploid (two clones), 2N-range aneuploid (two clones; clones 1 and 2 from Fig. 1C), and 4N-range aneuploid (two clones; +1q +2p −4q +7 ++8q −10p +10q +12 +20q −22 −X and −3q +5 ++8 +11 −15 −18 +20) cells treated with DHODHi with or without uridine rescue, as compared with control conditions for each line. Conditions shown are untreated, treated with 2 nM brequinar, and treated with 2 nM brequinar + 12 μM uridine. FDR values were calculated from edgeR-based analysis corrected for multiple hypothesis testing.

To profile the metabolic constraints that may lead to nucleotide pool insufficiency in aneuploid HMECs, we traced de novo pyrimidine synthesis flux into UTP using ^13^C_4_-labeled aspartate. This revealed that net gain aneuploid HMECs do not increase pyrimidine synthesis capacity, displaying equal label incorporation into UTP compared with diploids (Fig. 3A,B). However, because aneuploid HMECs have increased DNA content, this equivalent flux of de novo nucleotide synthesis compared with diploid cells represents a possible replication vulnerability, given that pyrimidine synthesis to ploidy ratios are effectively decreased (Fig. 3C–E). Additionally, we observed a significant decrease in GSH::GSSG ratios, indicating a disruption of redox balance and increased reactive oxygen species in aneuploid HMECs (Fig. 3F).

The underlying insufficiency in de novo pyrimidine flux relative to nucleotide demand suggests a mechanism for the sensitivity of aneuploid HMECs to DHODH disruption, which would exacerbate this pre-existing insufficiency. We thus examined the underlying metabolic disruptions induced by low-dose DHODHi (2 nM brequinar) in aneuploid and diploid cells using LC-MS/MS metabolomics. As expected, a buildup of the DHODH substrate dihydroorotate (DHO) was observed with DHODHi, validating the on-target nature of the drug (Fig. 3G). Diploid cells displayed minimal metabolomic changes associated with low-dose DHODHi, primarily a decrease in pyrimidine species, which were restored to baseline levels with uridine supplementation (Fig. 3H,I; Supplemental Fig. S3; Supplemental Table S6). However, 2N-range and 4N-range net gain aneuploid cells exhibit greater metabolomic disruption with DHODHi beyond just a decrease in pyrimidine species (which were also more significantly decreased than in diploids) (Fig. 3I; Supplemental Fig. S3). Furthermore, in aneuploid HMECs, uridine supplementation rescues most (but not all) of the metabolic disruption caused by DHODHi (Fig. 3I; Supplemental Fig. S3).

### Both oxidative phosphorylation and glycolytic rates are increased in net gain aneuploid HMECs

**Figure 4.**
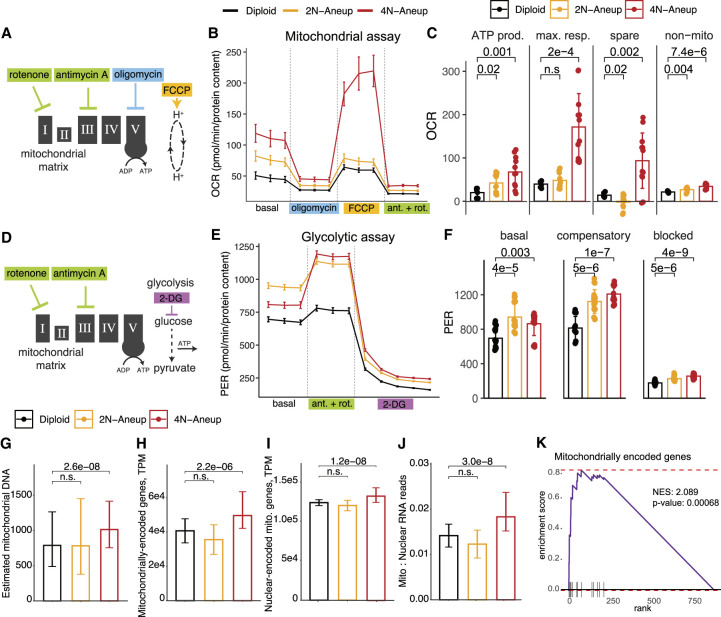
Net gain aneuploid HMECs exhibit increased mitochondrially linked ATP production and increased glycolytic rate. (*A*) Diagram depicting the point of action of the drugs used in the mitochondrial stress test assay. (*B*) Oxygen consumption rate (OCR) of diploid, 2N-range aneuploid, and 4N-range aneuploid cells after treatment with each of the drugs in the assay. The cell lines used in this experiment were the same as those described in Figure 3. (*C*) Bar graphs summarizing average baseline ATP production, maximal respiration, spare capacity, and nonmitochondrial respiration in each cell type. *P*-values were derived from *t*-tests and corrected for multiple hypotheses. (*D*) Diagram depicting the point of action of the drugs used in the glycolytic rate test. (*E*) Proton efflux rate (PER) of diploid, 2N-range aneuploid, and 4N-range aneuploid cells after treatment with each of the drugs in the assay. (*F*) Bar graphs summarizing average normalized PER levels during basal, compensatory, and blocked phases of the assay in diploid, 2N-range aneuploid, and 4N-range aneuploid cells. *P*-values were derived from *t*-tests and corrected for multiple hypotheses. (*G*–*J*) Bar graphs showing the estimated number of mitochondrial DNA copies relative to nuclear DNA content (*G*), the transcripts per million (TPM) of mitochondrially encoded genes (*H*), the TPM of nuclear-encoded mitochondrial genes (*I*), and the ratio of mitochondrial to nuclear RNA reads (*J*) in diploid, 2N-range aneuploid, and 4N-range aneuploid cells. (*K*) Mitochondrially encoded gene set enrichment in 4N-range aneuploid HMECs compared with diploids. Enrichment score and *P*-value from the analysis are shown.

Because we also observed increased dependence of net gain aneuploid cells on mitochondrial pathways, including oxidative phosphorylation, and increased indicators of redox stress like GSH::GSSG ratio (Fig. 3F), we asked whether baseline bioenergetic states of aneuploid and diploid cells were altered. We found that aneuploidy increases oxygen consumption to a degree scaling with overall ploidy, whereas proton efflux rates also increase in aneuploid cells but do not scale in the same manner, indicative of generally increased oxidative phosphorylation and glycolytic flux, respectively (Fig. 4A–F; Supplemental Fig. S4A–D). We next assessed total mitochondrial content relative to DNA ploidy across a broad range of aneuploidies in HMECs. We found an increase in estimated mitochondrial DNA in 4N-range aneuploids (even relative to ploidy), along with an increase in both nuclear and mitochondrially encoded mitochondrial gene expression (Fig. 4G–K). Thus, mammary epithelial cells with higher chromosomal ploidies tend to increase mitochondrial content and mitochondrial gene expression and display higher rates of oxidative phosphorylation. The increased flux through oxidative phosphorylation and glycolysis observed in net gain aneuploids may serve to compensate for increased energy expenditure, because ratios of ATP::AMP are largely equivalent in aneuploids and diploids (Supplemental Fig. S4E).

### Net gain aneuploid HMECs activate p53 and are sensitive to DNA-damaging drugs combined with DHODHi

**Figure 5.**
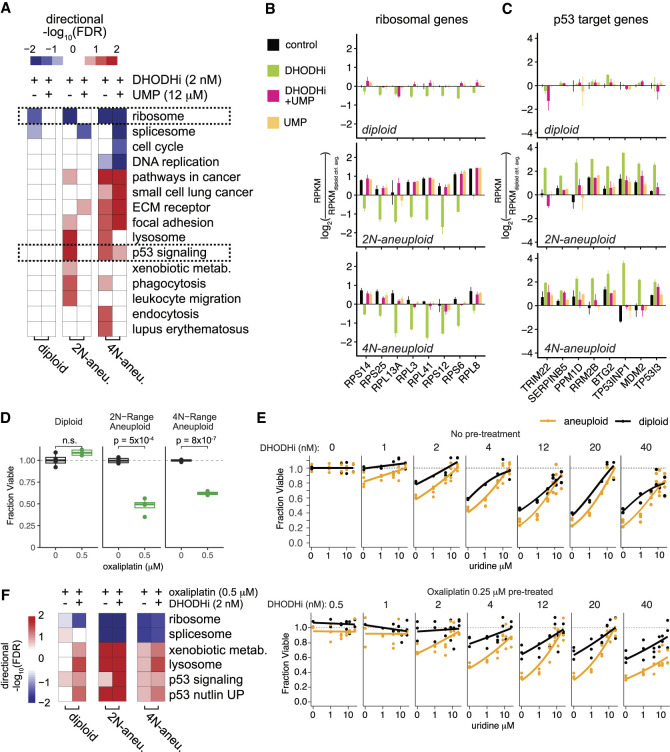
Nucleotide pool stress signaling is activated by low-dose DHODHi and DNA-damaging agents in net gain aneuploid HMECs. (*A*) Heat map summary of transcriptomic profiles of representative diploid, 2N-range aneuploid (clone 2), and 4N-range aneuploid (−3q +5 ++8 +11 −15 −18 +20) HMECs treated with the DHODH inhibitor brequinar or with a combination of brequinar and UMP, relative to control media conditions. Relative expression levels of ribosomal genes and p53 target genes are outlined with a dotted black box. (*B*,*C*) Bar graphs showing relative reads per kilobase per million reads (RPKM) of several ribosomal genes (*B*) and p53 target genes (*C*) in diploid, 2N-range aneuploid, and 4N-range aneuploid cells treated with brequinar, brequinar and UMP, or UMP or under control media conditions. (*D*) Cell viability of untreated diploid, 2N-range aneuploid, and 4N-range aneuploid HMECs treated with 0.5 μM oxaliplatin compared with control media conditions. *P*-values were calculated using *t*-tests. Representative HMEC clones are the same as those used in *A*. (*E*) Cell viability of diploid (shown in black) and 2N-range aneuploid HMECs (shown in yellow; combined data from clones 1 and 2) that have undergone either no pretreatment (*top*) or pretreatment with 0.25 μM oxaliplatin (*bottom*) and were subsequently challenged with 0–40 nM brequinar– with 0–10 μM uridine. (*F*) Heat map summary of transcriptomic profiles of representative diploid, 2N-range aneuploid (clone 2), and 4N-range aneuploid (+1q +2p −4q +7 ++8q −10p +10q +12 +20q −22 − X) HMECs treated with either oxaliplatin or a combination of oxaliplatin and brequinar, as compared with control conditions.

To assess the relationship between nucleotide pool insufficiency and stress signaling in aneuploidy, we performed RNA-seq in representative aneuploid and diploid HMEC lines under steady-state, mild nucleotide inhibition with DHODHi, or DHODHi plus UMP supplementation conditions. We validated that copy number-specific gene expression changes could be observed in this gene set, as expected (Supplemental Fig. S5A–D). Low-dose DHODHi (2 nM brequinar) resulted in decreased ribosomal gene expression universally, albeit to more significant degrees in aneuploid cells (Fig. 5A,B; Supplemental Table S7). Diploid transcriptomes were otherwise unperturbed by DHODHi, and the modest ribosomal gene downregulation was fully rescued by uridine salvage. 2N-range and 4N-range net gain aneuploid HMECs displayed more significant and widespread transcriptomic responses to DHODHi, including a robust activation of p53 signaling as indicated by canonical target upregulation (Fig. 5A,C), as well as activation of lysosomal, cell adhesion, and NFκB target genes (Supplemental Fig. S5E). Unlike in diploid cells, transcriptomic alterations in response to DHODHi were not fully restored to baseline with uridine supplementation in aneuploid HMECs (Fig. 5A).

We next assessed whether aneuploidy-associated nucleotide pool stress may result in sensitivity to chemotherapeutic DNA-damaging agents, which would exacerbate replication stress and nucleotide pool demand to support DNA repair. We exposed cells to 0.5 μM oxaliplatin for 3 days and found that aneuploid cells were significantly more sensitive to chemotherapeutic challenge than diploid cells (Fig. 5D). To test the combination of both DNA-damaging and DHODHi drugs, we pretreated cells with 0.25 μM oxaliplatin for 3 days to induce damage and then inhibited pyrimidine biosynthesis across a range of DHODHi doses and uridine rescue conditions. Pretreatment with oxaliplatin exacerbated the differential proliferative effects of DHODHi in aneuploid cells compared with diploid cells across a range of salvage conditions but especially when salvage is restricted by low uridine levels (Fig. 5E). Additionally, 0.5 μM oxaliplatin treatment for 3 days resulted in a transcriptional response similar to that observed for DHODHi-induced nucleotide pool stress, including downregulation of ribosomal gene expression and increased p53 signaling in aneuploid cells, and this response was even more pronounced when treatments were combined (Fig. 5F).

### Net gain aneuploid tumors and cancer cell lines display similar metabolic features

**Figure 6.**
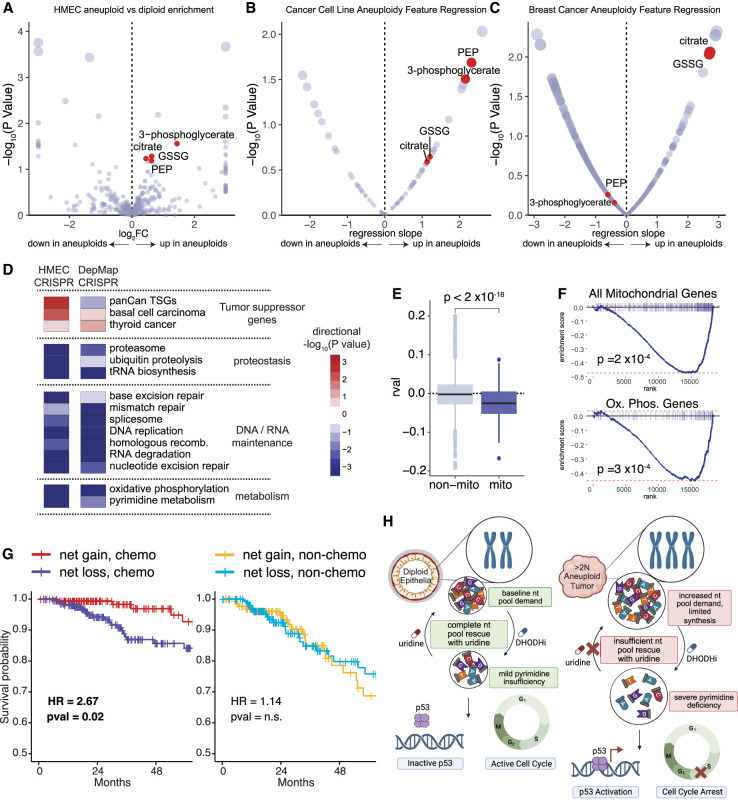
Net gain aneuploidy is associated with metabolic phenotypes and is prognostic for response to DNA-damaging chemotherapy in cancer. (*A*) Volcano plot showing metabolite log_2_ fold change (log_2_FC) (*X*-axis) and −log_10_(*P*-value) (*Y*-axis) in net gain aneuploid HMECs compared with diploid HMEC controls under steady-state conditions. Log_2_FC capped at minimum −3 and maximum 3. (*B*) Volcano plot showing linear regression coefficient (*X*-axis) and corresponding −log_10_(*P*-value) (*Y*-axis) of metabolite levels compared with net gain aneuploidy levels across cancer cell lines. (*C*) Volcano plot showing linear regression coefficient (*X*-axis) and corresponding −log_10_(*P*-value) (*Y*-axis) of metabolite levels compared with net gain aneuploidy levels across a cohort of human breast cancer samples (aneuploidy levels inferred from matched RNA-seq data). (*D*) Heat map of differentially essential gene sets associated with net gain aneuploidy across DepMap cancer cell line CRISPR screen data (*right* column) showing a pattern similar to that of the aneuploidy epistasis profile of HMECs (*left* column). Gene set enrichment analysis (KEGG gene sets) was performed on a gene list ranked by the correlation of effect scores with net gain aneuploidy levels. HMEC data are summarized from CRISPR screens in Figure 1. (*E*) Gene effect scores of mitochondrially localized genes are mostly negatively correlated with net gain aneuploidy levels across cancer cell lines. (*F*) Gene set enrichment plots showing all mitochondrially localized genes (*top*) or just oxidative phosphorylation genes (*bottom*) using the gene ranking in *D*. (*G*) Overall survival across a 5 year time frame of breast cancer patients from the TCGA cohort with aneuploid tumors classified as either “net gain” or “net loss,” treated with DNA-damaging/antinucleotide/antimetabolite chemotherapeutics (*left* panel) or without chemotherapy (*right* panel). Hazard ratios and associated *P*-values were calculated from Cox proportional hazards regression models. (*H*) Diagram depicting a net gain aneuploidy nucleotide insufficiency model and putative therapeutic vulnerability. Increased nucleotide pool requirements render net gain aneuploid cells sensitive to nucleotide pool stress caused by nucleotide synthesis inhibitors or DNA damage. This nucleotide pool stress cannot be fully rescued by uridine salvage and results in p53 activation and cell cycle arrest, whereas diploid cells can replicate efficiently using salvage alone.

To determine whether aneuploidy-associated nucleotide insufficiency phenotypes were also present in human tumors and cancer cell lines, we assessed large-scale metabolomics, sequencing, and functional genomics data sets. A paired RNA-seq/metabolomics data set of 108 breast carcinoma samples with matched normal tissue (Xiao et al. 2022) enabled us to infer copy number profiles of tumors (Supplemental Fig. S6A) and assess metabolic trends associated with net gain tumors. We performed a similar analysis of 907 cancer cell lines (pan-subtype) with paired copy number and metabolomics data (Ghandi et al. 2019; Li et al. 2019). Several metabolic shifts that were observed in net gain aneuploid HMECs relative to diploid HMECs at steady state, including increased TCA cycle intermediates (citrate), oxidized glutathione (GSSG), and phosphoenolpyruvate (PEP), were also observed in net gain cancer cell lines and breast carcinoma samples (Fig. 6A–C; Supplemental Fig. S6B–D). This may indicate increased TCA cycle/oxidative phosphorylation activity in addition to increased glycolysis rates associated with net chromosomal gain, as confirmed with bioenergetic assays in HMECs (Fig. 4).

To determine what genetic dependencies correlated with net gain aneuploidy broadly across cancer cell lines, we assessed the DepMap CRISPR data (Tsherniak et al. 2017) along with chromosomal copy number data across 756 cancer cell lines (Supplemental Table S8). Ranking gene effects by their correlation to the degree of net gain aneuploidy in cancer cell line genomes and performing gene set enrichment analysis revealed a similar pathway-level synthetic lethality profile compared with the HMEC aneuploidy synthetic lethality screens in this study (Fig. 6D; Supplemental Fig. S6E). The top synthetic lethal pathways with net gain aneuploidy in cancer cell lines included oxidative phosphorylation, pyrimidine metabolism, DNA repair pathways, and proteostasis pathways (Fig. 6D; Supplemental Fig. S6E). The essentiality of genes encoding mitochondrially localized proteins was generally correlated with the degree of net gain aneuploidy across cancer cell lines (Fig. 6E,F). A similar overall gene set enrichment profile was observed when analyzing net gain aneuploidy in just breast cancer-derived cell lines (*n* = 33) (Supplemental Fig. S6F). This indicates that net gain aneuploidy universally confers sensitivity to disruption of nucleotide, DNA repair, and mitochondrial pathways in both non-cancer-derived and cancer-derived cell line models.

### Breast cancer patients with net gain aneuploid tumors have increased survival probability when treated with DNA-damaging chemotherapies

These combined functional genomics analyses suggest that nucleotide insufficiency in net gain aneuploid tumors may represent a therapeutically exploitable vulnerability to drugs that affect the nucleotide pool and DNA replication, such as DNA-damaging chemotherapeutics (like platin-based therapies), nucleotide analogs (like 5-FU), or antimetabolites (like methotrexate). Indeed, stratifying patients in the breast cancer TCGA cohort by aneuploidy (net gain vs. net loss) and treatment type (chemotherapy vs. nonchemotherapy) revealed that patients with net gain tumors treated with chemotherapy have a significantly higher survival probability than patients with net loss tumors (HR = 2.67, *P* = 0.02) (Fig. 6G). This difference in survival was not observed in patients who were treated with nonchemotherapeutic hormonal and/or targeted therapies (Fig. 6G). The best overall survival was achieved in the net gain aneuploidy and chemotherapy treatment group (95% 5 year survival rate) (Fig. 6G). This suggests that aneuploidy status could be used in therapeutic decision-making and patient stratification and that patients with net gain aneuploid tumors could benefit significantly from receiving DNA-damaging/nucleotide analog/antimetabolite chemotherapies. Incorporating biosynthesis inhibitors like DHODHi into treatment regimens may provide additional benefit by exploiting the inability of net gain aneuploid cells to proliferate using pyrimidine salvage alone, with minimal uridine supplementation to buffer systemic toxicity while still restricting aneuploid tumor cell growth (Fig. 6H).

## Discussion

In this study, we performed genome-wide CRISPR knockout screens in human mammary epithelial cells with net gain aneuploidy compared with isogenic diploid cells. In addition to known aneuploidy stress-related susceptibilities like ubiquitin-mediated protein homeostasis, we identified mitochondrial oxidative phosphorylation, TCA cycle, and de novo pyrimidine biosynthesis enzymes as top-scoring dropout hits in the screen. Additionally, we observed increased enrichment of sgRNAs targeting components of the p53 pathway in aneuploid cells compared with diploids, indicating that aneuploid cells derive greater benefit from loss of commonly mutated tumor suppressors. Aneuploidy has been shown to activate p53 signaling in multiple cell types (Burrell et al. 2013; Ohashi et al. 2015; Santaguida et al. 2017; Zhu et al. 2018), including HMECs (Fig. 5A,B; Watson et al. 2024). Additionally, loss of p53 via mutation licenses aneuploidy in vitro (Li et al. 2010) and is associated with increased aneuploidy in human tumors (Ciriello et al. 2013; Taylor et al. 2018). Replication stress has been previously proposed as a mechanism linking aneuploidy and p53 activation (Burrell et al. 2013); however, the root causes of aneuploidy-associated replication stress are not well understood. Our study reveals that nucleotide pool insufficiency is a metabolic vulnerability of net gain aneuploid HMECs, which is associated with p53 activation and is exploitable with pyrimidine synthesis inhibitors.

DHODH appears to be a key node linking increased reliance on mitochondria and nucleotide metabolism in aneuploid cells. DHODH is the sole mitochondrial-resident enzyme of the de novo pyrimidine biosynthesis pathway, which reduces ubiquinone in the dehydrogenation of dihydroorotate to orotate. Although we observed significantly increased ATP production via oxidative phosphorylation as well as increased glycolysis rates in net gain aneuploids, we did not observe increased flux through de novo pyrimidine biosynthesis, perhaps revealing why it is the top vulnerability of aneuploid HMECs. Sensitivity to DHODH inhibition in net gain aneuploid cells may ultimately be due to a mismatch between nucleotide synthesis rates and nucleotide demand. Knocking out *DHODH* with CRISPR revealed that, although diploid HMECs could proliferate using solely de novo pyrimidine synthesis or salvage with the ability to switch seamlessly between the two, aneuploid HMEC proliferation was significantly impaired under the salvage-only condition. This indicates a constitutive reliance on de novo pyrimidine synthesis in mammary epithelial cells with extra chromosomes even when nucleotides are plentifully available for salvage, perhaps due to a fixed upper limit on import or salvage capacity. This is supported by the observation that high levels of supplemented uridine failed to compensate for pyrimidine synthesis deficiency in net gain aneuploids (Fig. 2C).

Mild impairment of DHODH function with low-dose brequinar treatment exacerbated nucleotide pool stress and metabolome disruption (Fig. 3), as well as the underlying p53 activation phenotype associated with aneuploidy in HMECs (Fig. 5). Interestingly, ribosomal gene expression is increased at baseline in aneuploid cells relative to diploids and then decreases dramatically in response to DHODHi (Fig. 5B, black bars). Ribosome biogenesis could be expected to compete with DNA replication for nucleotide pools given that the total rRNA content in a human cell (∼8–16 pg) is greater than the total amount of genomic DNA (∼6 pg) (Han and Lillard 2000); thus, the interplay between these major consumers of pyrimidines could form an intersection of vulnerability in net gain aneuploid cells. Activation of p53 occurs in response to both rRNA/ribosome biogenesis disruption and replication stress (Lindström et al. 2022), again highlighting the central role of p53 in aneuploidy stress sensing and response.

Combination treatment regimens revealed that pretreatment with DNA damagers increases cell sensitivity to DHODH inhibition, particularly in aneuploid cells. These data suggest the potential for adjuvant therapies using lower doses of nucleotide inhibitors in combination with other clinically available DNA-damaging chemotherapies, which have been shown to increase reliance on de novo pyrimidine synthesis in breast cancer (Brown et al. 2017). Analysis of survival data across a large cohort of breast cancer patients suggests that net gain aneuploidy could be used as a biomarker to identify patients who are likely to respond to DNA-damaging and antimetabolite/antinucleotide treatments (Fig. 6). Physiological concentrations of uridine are more efficient at rescuing pyrimidine synthesis deficiency in diploid cells compared with aneuploid cells, suggesting a potential window for limited nucleotide supplementation to be used in conjunction with nucleotide inhibitors to titrate drug toxicity in healthy tissue while still maintaining efficacy in tumors. Nucleotide salvage should be further investigated as a targetable node, as has been explored in kidney cancers with FH mutation and subsequent reliance on purine salvage (Wilde et al. 2023). Because many tissues can access both synthesis and salvage of nucleotides (Tran et al. 2024), further exploration of the generalizability of our findings to net gain aneuploidy in other tumors types is warranted.

In summary, mammary epithelial cells with excess chromosomes experience nucleotide pool stress due to the increased demand for, but insufficient compensatory synthesis/salvage of, pyrimidines. Furthermore, p53 activation and stress signaling in net gain aneuploid mammary cells may result from intrinsic metabolic constraints on pyrimidine synthesis and salvage leading to replication stress, which can be further exacerbated by pyrimidine synthesis inhibitors or DNA damage. These results have wide-ranging implications for cancer treatment strategies, as well as metabolic management of aneuploidy-associated syndromes such as Down syndrome and sex chromosome abnormalities.

## Materials and methods

### Cell culture

The hTERT HMEC line was immortalized previously (Solimini et al. 2012) from primary HMECs purchased from ATCC (PCS-600-010). Low-passage hTERT HMECs were grown in Lonza HMEC medium with bovine pituitary extract and growth supplements. Cells were passaged once they reached ∼90% confluency using 5 mL of 0.05% trypsin. We previously confirmed the identity of this cell line using DNA-seq and RNA-seq analysis (Watson et al. 2024). Human embryonic kidney (HEK) 293T cells used for lentiviral production were cultured in DMEM supplemented with 10% FBS and 1% penicillin/streptomycin.

### Genome-wide CRISPR screens

A genome-wide CRISPR library targeting ∼18,000 genes with five sgRNAs per gene was used in the lentiCRISPR v2 backbone. The library was packaged along with lentiviral packaging components (Tat, Rev, Gagpol, and Vsvg) into lentiviral particles using lipofectamine transfection of HEK293T cells in lentiviral packaging media (Opti-MEM reduced serum media with 5% FBS, 1× GlutaMAX supplement). Lentivirus was harvested and concentrated using Lenti-X concentrator. Target HMECs were infected with the library at a multiplicity of infection (MOI) of 0.3 to ensure single viral integration per cell. A representation of 500× was maintained throughout the experiment to ensure robust screening coverage. Following infection, HMECs were selected with puromycin at a concentration of 2 μg/mL for 2 days to enrich for cells that were successfully transduced with the lentiviral constructs. After selection, cells were expanded in culture and harvested at two time points: immediately after puromycin selection (time point PD0) and after the cells had undergone approximately six population doublings (PD6). Genomic DNA was isolated from the collected cell pellets after lysis with proteinase K treatment using the phenol–chloroform extraction method. The regions containing the integrated sgRNA sequences were amplified by PCR to generate amplicons for sequencing. Amplicon sequencing was performed on the Illumina NextSeq 550 platform to quantify the abundance of each sgRNA construct in the cell populations. Sequencing reads were aligned to the reference library using Burrows–Wheeler aligner (BWA) (Li and Durbin 2009). The differential depletion or enrichment of sgRNAs between PD0 and PD6 was analyzed using the limma–voom pipeline in conjunction with edgeR (Robinson et al. 2010; Ritchie et al. 2014) and the camera (Wu and Smyth 2012) function. Gene set enrichment analysis was performed using the fgsea (Korotkevich et al. 2021) package to identify significantly enriched pathways or gene sets.

### Generating DHODH^−/−^ cell lines with CRISPR

The lentiCRISPR v2 backbone (Sanjana et al. 2014) was digested with the BsmBI restriction enzyme, and the cut vector was isolated through a gel extraction. sgRNA guides targeting *DHODH* were then ligated into the digested lentiCRISPR v2 backbone and packaged into lentivirus through transfection of HEK293T cells along with lentiviral packaging components (Tat, Rev, Gag/Pol, and Vsvg) with Lipofectamine 3000 in lentiviral packaging media made of Opti-MEM reduced serum media with 5% FBS and 1× GlutaMAX supplement. Lentivirus was then harvested and passed through a 0.45 μm filter and used to transduce HMECs. Transduced cells were then selected with 2 μg/mL puromycin for 2 days and recovered in HMEC media as described above, supplemented with 20 μM uridine. The reduction in levels of DHODH protein was quantified through Western blots (see below). The sgRNA target sequence for the control *AAVS1* locus was GGGGCCACTAGGGACAGGAT, and the sgRNA target sequence for *DHODH* was GTGTTCGCTTCACCTCCCTG.

### Western blots

Cell pellets of ∼5 × 10^5^ cells from each HMEC line were lysed in 250 μL of 2× RIPA buffer plus protease inhibitor cocktail. Lysates were vortexed and spun down, protein concentrations were determined by bicinchoninic acid protein assay (Pierce 23227), and equal amounts of protein were mixed with Pierce lane marker reducing sample buffer and loaded onto 1.5 mM 4%–12% Bis-Tris gels with 15 wells (Invitrogen NP0336BOX). Gels were run in MOPS SDS buffer (Life Technologies NP0001), transferred to nitrocellulose (Bio-Rad 170-4158), blocked overnight in 3% BSA at 4°C, and incubated overnight at 4°C with DHODH antibody (DHODH [E9X8R] rabbit mAb; Cell Signaling 26381S) at 1/1000 dilution in TBST buffer with 1% BSA or with vinculin antibody (vinculin [EPR19579] rabbit mAb; Abcam ab207440) at 1/1000 dilution. Secondary antibody for all assays was goat antirabbit IgG (Abcam ab205718) with incubation at 1/10,000 dilution for 1 h at room temperature. Western blots were quantified using ImageJ v1.53a (Schneider et al. 2012).

### Cell viability assays

HMECs of varying degrees of aneuploidy (see the figure legends for specific karyotypes corresponding to each experiment) were seeded at a concentration of 5 × 10^5^ cells/mL in a final volume of 0.1 mL in 96 well flat-bottom microtiter plates. We used the fluorometric resazurin reduction method (CellTiter-Blue, Promega) to evaluate the chemosensitivity of cells treated with brequinar and rescued with uridine (Sigma Chemical Co.). These drugs were added in a range of 1–12 nM or 1–12 μM, respectively, and cells were treated for 48 h. Fluorescence (537 Ex/610 Em) was determined using a luminometer (Tecan microplate reader). The percentage of viable cells was calculated using modeling technique.

### PI staining for total DNA content

Approximately 5 × 10^5^ cells per clone were fixed in 70% ethanol and then stored for up to 1 month at −20°C. Fixed cells were spun down, fixative was removed, and cells were washed once in phosphate-buffered saline (PBS) and finally resuspended in 500 μL of Thermo Fisher FxCycle PI/RNase staining solution. After incubation in the dark for 30 min, cells were passed through a mesh filter sieve and analyzed by fluorescence-activated cell sorting (FACS) using 532 nm excitation with a 585/42 nm bandpass filter. An average of 1 × 10^4^ events was analyzed per clone, with data collected via BD FACSDiva software v.8.0 and processed using R packages flowCore (Hahne et al. 2009) and ggcyto (Van et al. 2018) to derive the average fluorescence of the G1 peak relative to that of diploid control cells processed simultaneously.

### RNA-seq library preparation and analysis

A total of 5 × 10^5^ cells from each cell line was plated in 10 cm plates and grown for 144 h. Cells were provided fresh media 3 h before collection. Media were aspirated, cells were immediately lysed in dishes, and total RNA was purified using Qiagen RNeasy kits. A quantity of 1 μg of total RNA was used for mRNA purification with the NEBNext poly(A) mRNA magnetic isolation module. NEBNext Ultra II directional RNA library preparation kits for Illumina were used for RNA-seq library preparation. NEBNext multiplex oligos for Illumina were used for indexing during PCR amplification of the final libraries. Libraries were quantified by qPCR using the NEBNext library quantification kit for Illumina and multiplexed accordingly. RNA-seq reads were aligned to the human reference genome hg37 using BWA (Li and Durbin 2009). Following alignment, SAMtools (Li et al. 2009) was used to sort the aligned reads, which were then compiled into gene-level read counts using the subread featureCounts (Liao et al. 2014) function. Gene-level differential expression was analyzed using the edgeR (Robinson et al. 2010; Ritchie et al. 2014) limma function, and differential pathway analysis was performed with fgsea using KEGG (Kanehisa and Goto 2000) gene sets.

### LC-MS/MS metabolomic profiling, aspartate tracing, and analysis

#### Polar LC-MS method

A Q-Exactive orbitrap mass spectrometer with an Ion Max source and HESI II probe attached to a Vanquish Horizon UHLPC system was used to measure polar metabolites. The LC-MS underwent weekly cleaning and calibration with positive and negative Pierce ESI ion calibration Calmix (Thermo Scientific). Two microliters of sample was injected into the machine and run through a SeQuant ZIC-pHILIC 5 μm 150 mm × 2.1 mm analytical column (Sigma) with a 2.1 mm × 20 mm guard column (Sigma) attached to the front end. The column oven was set to 25°C and the autosampler was set to 4°C. Buffer A was comprised of 20 mM ammonium carbonate (Sigma) and 0.1% ammonium hydroxide (Sigma) in HPLC-grade water (Sigma), and buffer B was comprised of 100% acetonitrile (Sigma). The liquid chromatography was set to a flow rate of 0.15 mL/min. First, a linear gradient from 80% buffer B to 20% buffer B occurred over the course of 20 min, followed by a linear gradient from 20% buffer B to 80% buffer B for 0.5 min, and then a hold at 80% buffer B for 7.5 min. The mass spectrometer was set to full scan (70–1000 *m*/*z*) and polarity switching mode, with the spray voltage set to 4.0 kV, heated capillary set to 350°C, and the HESI probe set at 30°C. The sheath gas flow was set at 10 U, auxiliary gas was set at 1 U, and sweep gas flow was set at 1 U. The resolution of scan was set to 70,000, AGC target was set to 1 × 10^6^, and maximum injection time was set at 20 msec. An additional scan between 220 and 700 *m*/*z* was used to enhance nucleotide detection in the negative mode as well with the maximum injection time set to 80 msec.

#### Polar metabolite isolation from cultured cells

Media were aspirated from the plates and then the cells were washed twice with 1× PBS. The plate was then transferred to dry ice, and 500 µL of 80% HPLC-grade methanol (Sigma)/20% HPLC-grade water (Sigma) was added to each well. The wells were placed in a −80°C freezer to incubate for 15 min. The plates are taken out of the freezer one at a time and placed back on dry ice. The cells were then scraped and transferred to a new tube. Each well was washed with an additional 300 µL of 80% HPLC-grade methanol (Sigma)/20% HPLC-grade water (Sigma) and collected into the same tube as the initial lysis. The samples were then vortexed for 10 min at 4°C and centrifuged at 21,300*g* for 10 min at 4°C. Supernatants were transferred to a new tube and dried down in a refrigerated CentriVap benchtop vacuum concentrator connected to a CentriVap-105 cold trap (Labconco). After being dried down, pellets were stored in a −20°C freezer until ready to run on the polar LC-MS method.

#### ^13^C_4_-aspartate tracing in vitro

Cells were seeded in Lonza HMEC medium 48 h prior to tracing so that wells reached 75% confluence at the time of the experiment. Six hours prior to metabolite isolation, the cells were treated with Lonza HMEC medium containing 10 mM ^13^C_4_-aspartate (Sigma), and the pH was adjusted to 7.4 with the relevant treatments. After the 6 h incubation period, polar metabolites were then isolated as described above and run on the polar LC-MS method.

#### LC-MS data analysis

Metabolomics data were analyzed using TraceFinder 5.1 (Thermo Fisher). Peaks were integrated using a strict 5 ppm mass tolerance and attention to the retention times as determined by purified standards of the respective metabolites. ^13^C- and ^15^N-isotopologs were integrated with the same retention time as the ^12^C- and ^14^N-isotopologs. All stable isotope tracing data underwent natural abundance correction using IsoCorrectoR (Heinrich et al. 2018).

### Seahorse

Mitochondrial respiration and glycolytic rate of HMECs was measured as their oxygen consumption rate (OCR) and extracellular acidification rate (ECAR), respectively, using an oxygen-controlled XFe96 extracellular flux analyzer (Seahorse Bioscience). Diploid, 2N-range cells, and 4N-range cells were seeded in 12 replicates at 1 × 10^5^ cells per well, 2 × 10^5^ cells per well, and 2.5 × 10^5^ cells per well, respectively, in 80 μL of Lonza media into XFe96 cell culture microplates (Agilent) 24 h prior to the experiment. Concurrently, the XFp sensor cartridge (Agilent) was hydrated by adding 200 μL of XF Calibrant (Agilent) and left in a CO_2_-free incubator overnight. One hour before the experiment, the Lonza media in the wells were replaced with 180 μL of Seahorse XF RPMI medium (Agilent) supplemented with 2 mM L-glutamine, 1 mM sodium pyruvate, and 10 mM D-glucose. After 1 h incubation in a CO_2_-free incubator at 37°C, glycolytic rate and mitochondrial stress tests were performed. Oxidative phosphorylation was assessed as follows using the oxygen consumption rate (OCR). Initially, the basal OCR was measured, followed by the addition of 2.5 μM oligomycin (Agilent), which inhibited complex 5 of the electron transport chain (ETC) and consequently decreased electron flow through the ETC. This allows for measurement of the ATP-linked respiration. Following this measurement, 2 μM carbonyl-cyanide-4 (triflouoromethoxy) phenylhydrazone (FCCP) (Agilent) was added, which was responsible for collapsing the proton gradient and disrupting the mitochondrial membrane potential, allowing for free flow of electrons through the ETC and measurement of the maximum mitochondrial respiration rate. The final injection included a combination of rotenone and antimycin A, which blocked complex 1 and complex 3, respectively. This resulted in complete inhibition of mitochondrial respiration, allowing for the nonmitochondrial respiration to be measured. Glycolytic rate was assessed by first measuring the basal proton efflux rate (PER). In this assay, the first injection included a 0.5 μM combination of rotenone and antimycin A to block mitochondrial respiration. The second injection, 50 mM 2-deoxy-D-glucose (2-DG), was then added, which competitively bound glucose-hexokinase and consequently inhibited glycolysis. The compensatory glycolytic rate was then measured by subtracting the PER after 2-DG injection from the PER after rotenone/antimycin A injection. Measurements were normalized using protein concentration obtained through the standardized protocol provided via the Thermo Scientific Pierce BCA protein assay kit.

### Nuclear:mitochondrial DNA content analysis

Previously acquired DNA sequencing data (Watson et al. 2024) were used to estimate mitochondrial DNA numbers relative to nuclear DNA content using fastMitoCalc (Qian et al. 2017).

### DepMap CRISPR screen analysis relative to copy number

Cancer cell line gene-level copy number information was used to rank cell lines by total net gain aneuploidy using a cutoff of log_2_(copy number ratio + 1) < 0.8 for losses and log_2_(copy number ratio + 1) > 1.16 for gains. DepMap CRISPR screen data gene effect scores were correlated to net gain aneuploidy levels across cell lines using linear regression analysis. Gene set enrichment analysis (Subramanian et al. 2005) using the KEGG gene sets (Kanehisa and Goto 2000) was used via the fgsea (Korotkevich et al. 2021) package in R to identify gene sets that were epistatic with net gain aneuploidy. To best compare with our screens, which were nucleotide supplement-free, we eliminated cancer cell lines from the analysis that were screened in media likely to contain nucleotide supplement (like F12 and medium 199) or for which media information was not available.

### Metabolomics analysis relative to copy number in cancer cell lines and tumors

Gene-level copy number data were used to determine net gain − loss values in each cancer cell line (Ghandi et al. 2019) using a cutoff log_2_(copy number ratio + 1) < 0.8 for losses and log_2_(copy number ratio + 1) > 1.16 for gains. Gain − loss values were then compared with metabolite levels (Li et al. 2019) across cell lines via linear regression analysis using the lm function in R.

For human tumor analysis, copy number was estimated across the genome from RNA-seq data for each tumor sample using the CreateInfercnvObject function in the inferCNV package in R. Normal (noncancerous tissue) samples from the same study were used as a reference group. Modified (normalized) expression was used to call gains or losses (>1.02 or <0.98, respectively) in genes likely attributable to copy number. Frequent breast cancer-associated events like +1q, −8p, +8q, and +16p could be observed in the inferCNV analysis, validating this methodology to detect cancer-associated CNAs from bulk RNA-seq data. Copy number calls from inferCNV were used to calculate gain − loss values for each tumor, which were then compared with metabolite levels via linear regression analysis using the lm function in R.

### Breast cancer survival analysis relative to copy number

Breast cancer copy number segment mean files generated by the TCGA Research Network (https://www.cancer.gov/tcga) were corrected for purity (Qin et al. 2018), and arm-level gain/loss calls were made based on purity-corrected log_2_(copy number ratio) < −0.41 for losses and purity-corrected log_2_(copy number ratio) > 0.32 for gains, with least 50% of the arm affected. Gain − loss values were calculated by subtracting the sum of total genomic megabases affected by chromosomal loss from the sum of total genomic megabases affected by chromosomal gain. Net gain tumors were annotated as those with gain − loss values >0, and net loss tumors were annotated as those with gain − loss values <0. Patients receiving DNA-damaging, antifolate, and antinucleotide chemotherapies were identified by the following search strings in the clinical data sets: “platin,” “dox,” “uracil,” “rubicin,” “mycin,” “phosphamid,” “citabine,” “cytoxan,” “trexate,” “ac,” “capecetabine,” “5-fu,” “metotreksat,” “mitoxantrone,” “cytoxen,” “gemzar,” “tc,” “tch,” and “xeloda.” The survival (version 2.11-4; https://github.com/therneau/survival) package in R was used for breast cancer patient survival analysis, using the Surv() and survfit() functions. Survival data were plotted using the ggsurvplot function from the survminer (version 0.4.9; https://github.com/kassambara/survminer) package in R. The coxph function in the survival package was used to fit a Cox proportional hazard regression model comparing outcomes in patients with net gain versus net loss tumors in chemo-treated and non-chemo-treated groups.

### Data Availability

All sequencing data are available on the NCBI SRA database, Bioproject ID PRJNA1165704.

## Supplemental Material

Supplement 1

Supplement 2

Supplement 3

Supplement 4

Supplement 5

Supplement 6

Supplement 7

Supplement 8

Supplement 9
