# Supplementary material for: Aneuploidy generates enhanced nucleotide dependency and sensitivity to metabolic perturbation
Source: Genes Dev. 2025 Jun 1;39(11-12):770–86. doi: 10.1101/gad.352512.124 (PMC12128873; doi:10.1101/gad.352512.124)
Supplement: Supplement 9 [file Supplemental_Figures_S1-S6.pdf]

Supplemental Figure 1

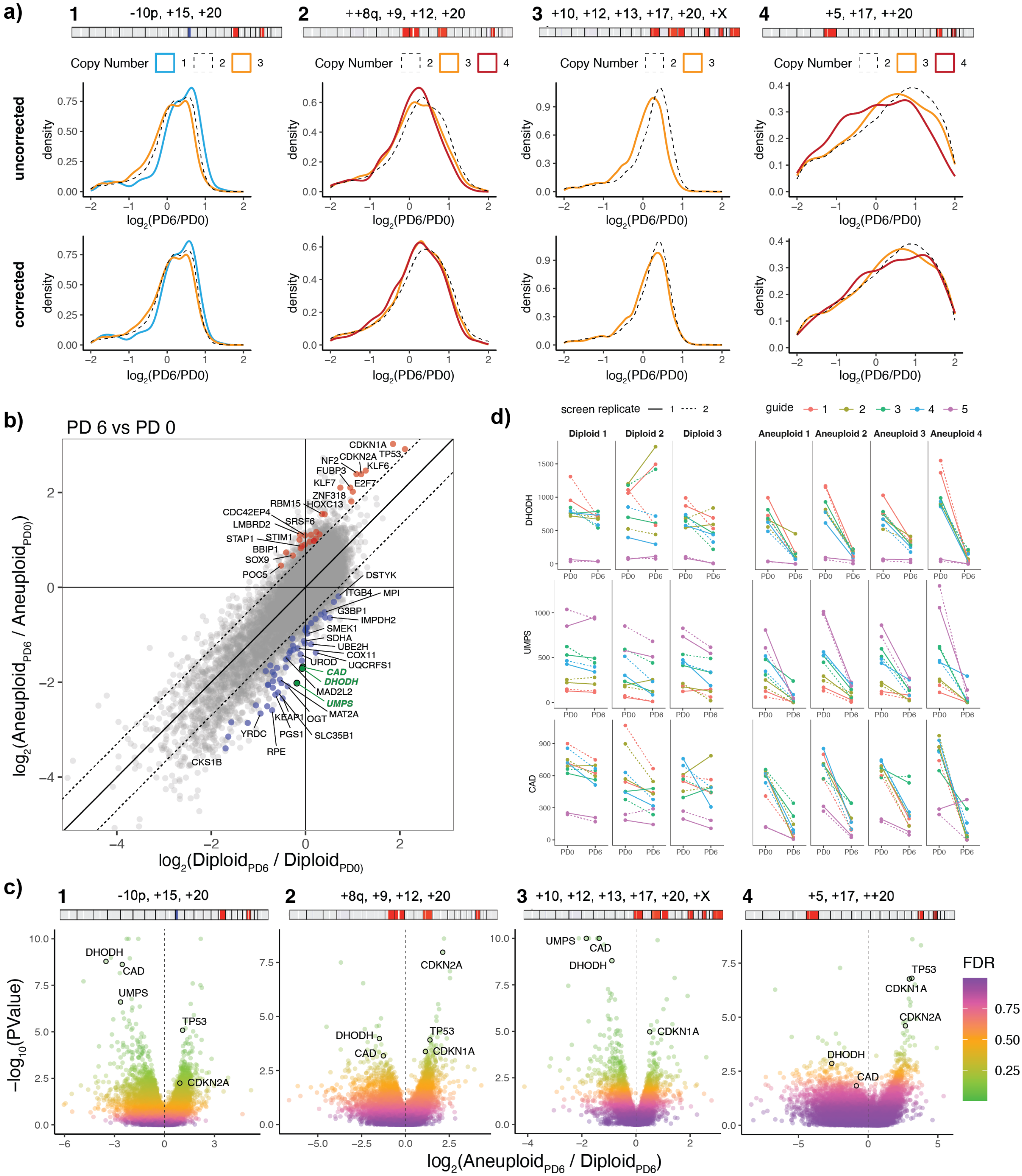

SUPPLEMENTAL FIGURE 1: Genome-wide CRISPR knockout screens in aneuploid and diploid HMECs

a, Distribution plots showing uncorrected (top) and copy number-corrected (bottom) CRISPR screen log_2(PD6/PD0) gene-level analysis, colored by gene ploidy level for each screen. Copy number profiles of aneuploid HMECs used in each screen are shown as colored bars above distribution plots. As previously reported[54], genes on gained chromosomes tend to exhibit more dropout, and genes on lost chromosomes tend to exhibit less dropout, due to the dosage of CRISPR-mediated DNA cutting. **b,** Gene-level dropout and enrichment comparing PD 6 (End) sgRNA levels to PD 0 (Start) sgRNA levels in diploid (x-axis) and aneuploid (y-axis) CRISPR screens. Genes that are differentially depleted (blue) and enriched (red) in aneuploid HMECs relative to diploid HMECs at PD 6 are shown. Pyrimidine biosynthesis genes CAD, DHODH, and UMPS are colored in green. **c,** Differential dropout/enrichment of gene-targeting sgRNAs in aneuploid clones at PD 6 compared to their isogenic diploid clones at PD 6. Bars indicating the karyotype profiles of each of the aneuploid clones are shown at the top of the figure. **d,** Individual sgRNA counts targeting DHODH, UMPS, and CAD in cell populations at PD 0 and PD 6 in each replicate screen diploid (left) and aneuploid (right) HMEC lines.

Supplemental Figure 2

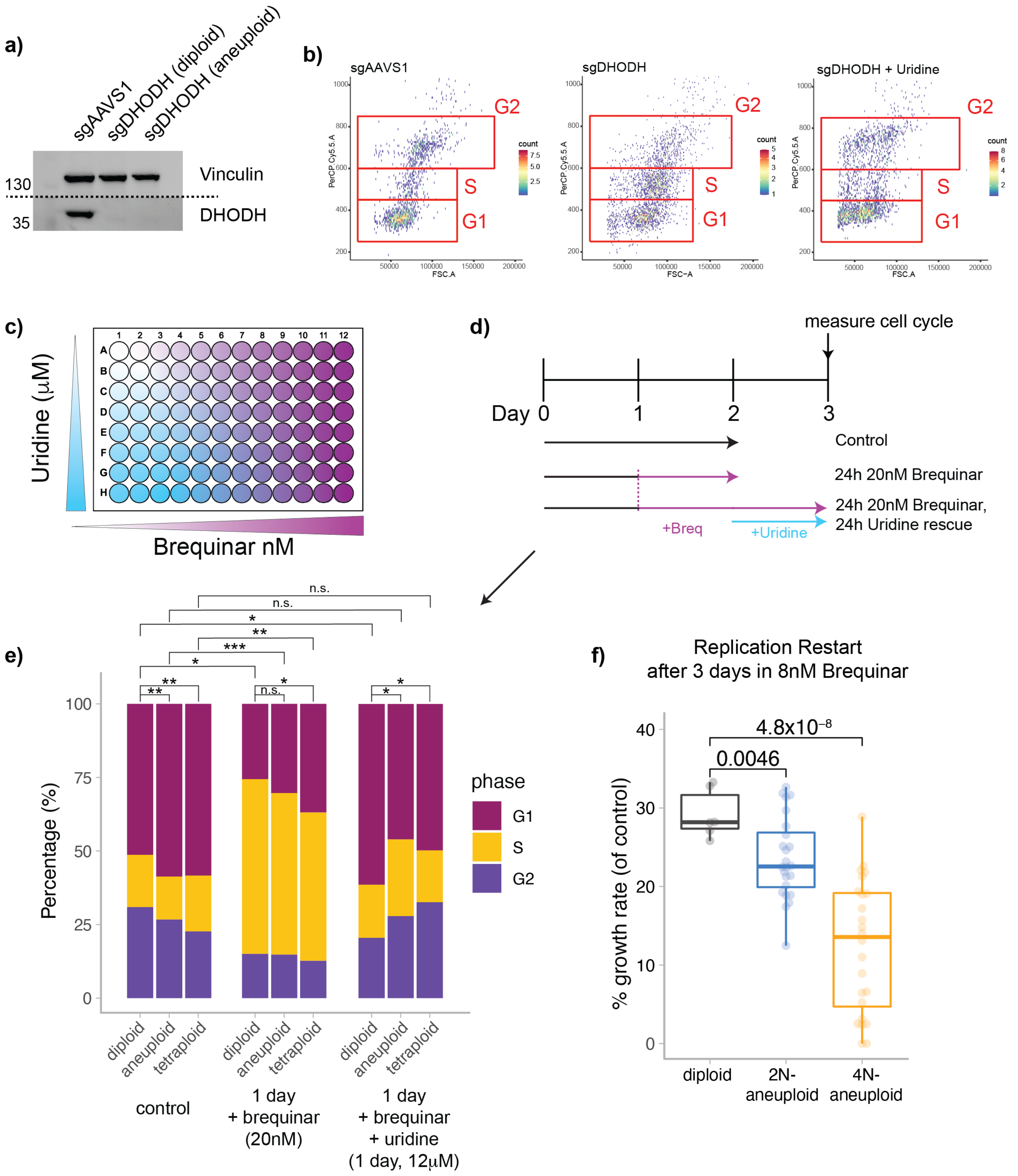

SUPPLEMENTAL FIGURE 2: Net-gain aneuploid HMECs are more sensitive to acute or prolonged disruption of pyrimidine biosynthesis.

a, Western blot showing complete knockout of DHODH in aneuploid and diploid cells exposed to DHODH-targeting sgRNA compared to an AAVS1-targeting control (in a diploid). DHODH knockout cells are maintained in uridine-supplemented media. These lines were used in the experiments in Fig. 2c. **b,** Flow cytometry data showing forward scatter as a proxy for cell size on the x-axis and propidium iodide staining fluorescence to quantify total DNA content on the y-axis. Gates indicate cells in G1, S, and G2 phases of the cell cycle. Related to Fig. 2b. **c,** Diagram showing the layout for the DHODHi (brequinar) + uridine supplementation matrix-style experiments in Fig 2e. **d,** Diagram showing the dosing schedule for control conditions (black), acute brequinar treatment (purple) and a combination of acute brequinar treatment (purple) and uridine (blue) for experiment shown in subsequent panel. **e,** Percentage of diploid, 2N-range aneuploid, and 4N-range aneuploid cells in G1, S, and G2 phase during pre-treatment, under experimental conditions described in **(d)**. P-values were calculated from wilcoxon rank tests corrected for multiple hypothesis testing, comparing percent of cells in S/G2 across cell types in each condition. The 2N-range aneuploid HMEC clones used in this experiment were clones #1-4 from Fig. 1c. The 4N-range aneuploid HMEC clones used in this experiment had the following karyotypes: (-3q -6 +8 +11 -15 +20), (+5 +13 +20 +X), and (+1q +2p -4q +7 ++8q -10p +10q +12 +20q -22 -X). **f,** The growth rate in diploid, 2N-range, and 4N-range aneuploid cells after DHODHi-mediated arrest for 3 days, followed by uridine rescue for 24 hours, represented as the percent of control condition growth rate. P-values were calculated from T tests corrected for multiple hypothesis testing. The 2N-range aneuploid HMEC clones used in this experiment were clones #1 and #2 from Fig. 1c. The 4N-range aneuploid HMEC clones used in this experiment were the same as those used in the previous panel.

Supplemental Figure 3

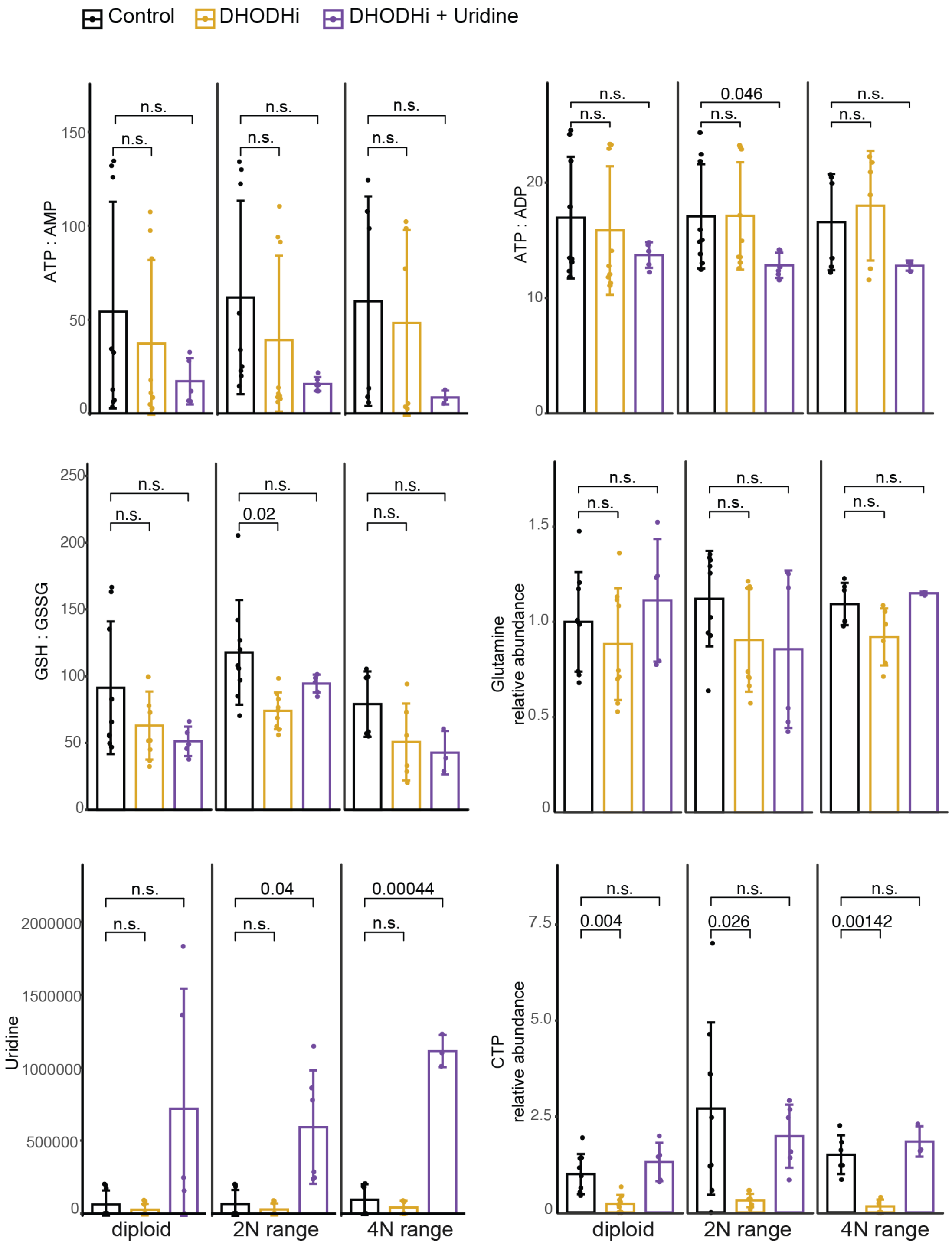

SUPPLEMENTAL FIGURE 3: Metabolites beyond just pyrimidines are disrupted by DHODH inhibition in net-gain aneuploid HMECs.

Bar graphs showing unchanged ratios of ATP::AMP or ATP::ADP metabolites and metabolite ratios in diploid, 2N-range aneuploid, and 4N-range aneuploid cells under control conditions, treatment with DHODH inhibitor, and treatment with DHODH inhibitor and uridine rescue. P-values were calculated using T tests. Related to Fig. 3i.

Supplemental Figure 4

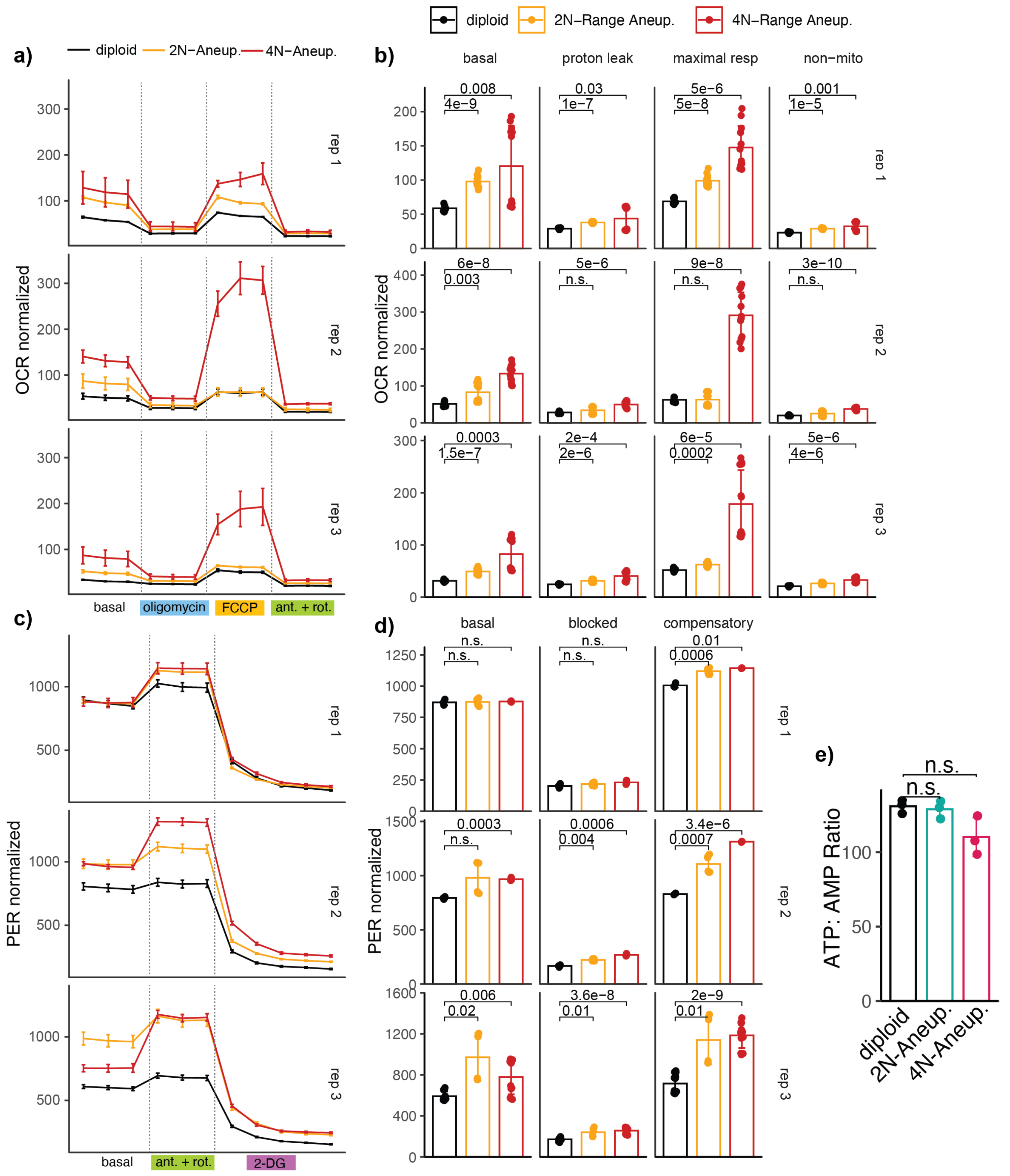

SUPPLEMENTAL FIGURE 4: Individual replicate experiments of mitochondrial stress test and glycolytic rate assays.

a, Oxygen consumption rates of three independent biological replicates of the Mitochondria Stress Test Assay using diploid, 2N-range aneuploid, and 4N-range aneuploid HMECs. Error bars indicate the standard deviation of technical replicate wells. Related to Fig. 4. **b,** Bar graphs showing the basal respiration, proton leak, maximal respiration, and non-mitochondrial respiration of diploid, 2n-range aneuploid, and 4n-range aneuploid HMECs across three replicates. P-values were calculated using T tests corrected for multiple hypothesis testing. **c,** Proton efflux rate of three independent biological replicates of the Glycolytic Rate Assay using diploid, 2N-range aneuploid, and 4N-range aneuploid HMECs. Error bars indicate the standard deviation of technical replicate wells. **d,** Bar graphs showing the basal, compensatory, and non-glycolytic proton efflux of diploid, 2n-range aneuploid, and 4n-range aneuploid HMECs across three replicates. P-values were calculated using T tests corrected for multiple hypothesis testing. **e,** Ratio of ATP:AMP in diploid, 2N-range, and 4N-range aneuploid HMECs under steady state conditions.

Supplemental Figure 5

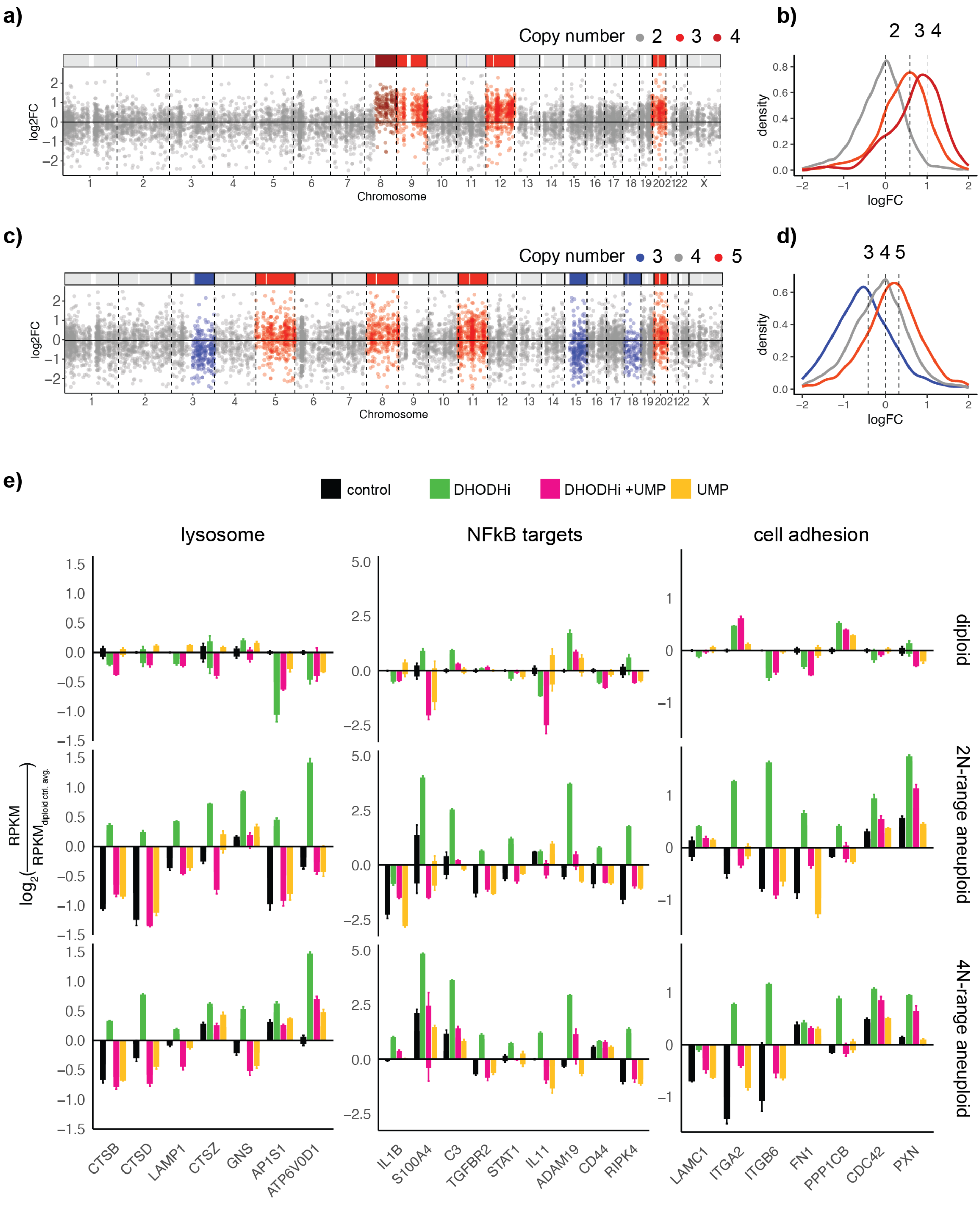

SUPPLEMENTAL FIGURE 5: Aneuploidy-dependent gene expression changes

a-d, As a validation of the dataset shown in Fig. 5 a-b, we observe expected copy number-dependent gene expression changes when comparing aneuploid to diploid control RNA-seq data. Gene expression log_2 fold change in a 2N-range aneuploid mutant relative to diploid controls plotted by genomic position **(a)** and as a density plot with respect to ploidy **(b)**. A similar genomic position plot **(c)** and density plot **(d)** for a 4N-range aneuploid HMEC line relative to diploid HMECs is shown. Copy number profiles for each line used are indicated by solid horizonal barplot across the top of each scatter plot. Genes are colored by gain/loss status. **e,** Bar graphs showing relative reads per kilobase per million reads of specific lysosome, NFkB, and cell adhesion genes in diploid, 2N-range aneuploid, and 4N-range aneuploid cells treated with the indicated conditions. These gene sets were upregulated in aneuploid cells but not diploid cells under DHODH inhibition.

Supplemental Figure 6

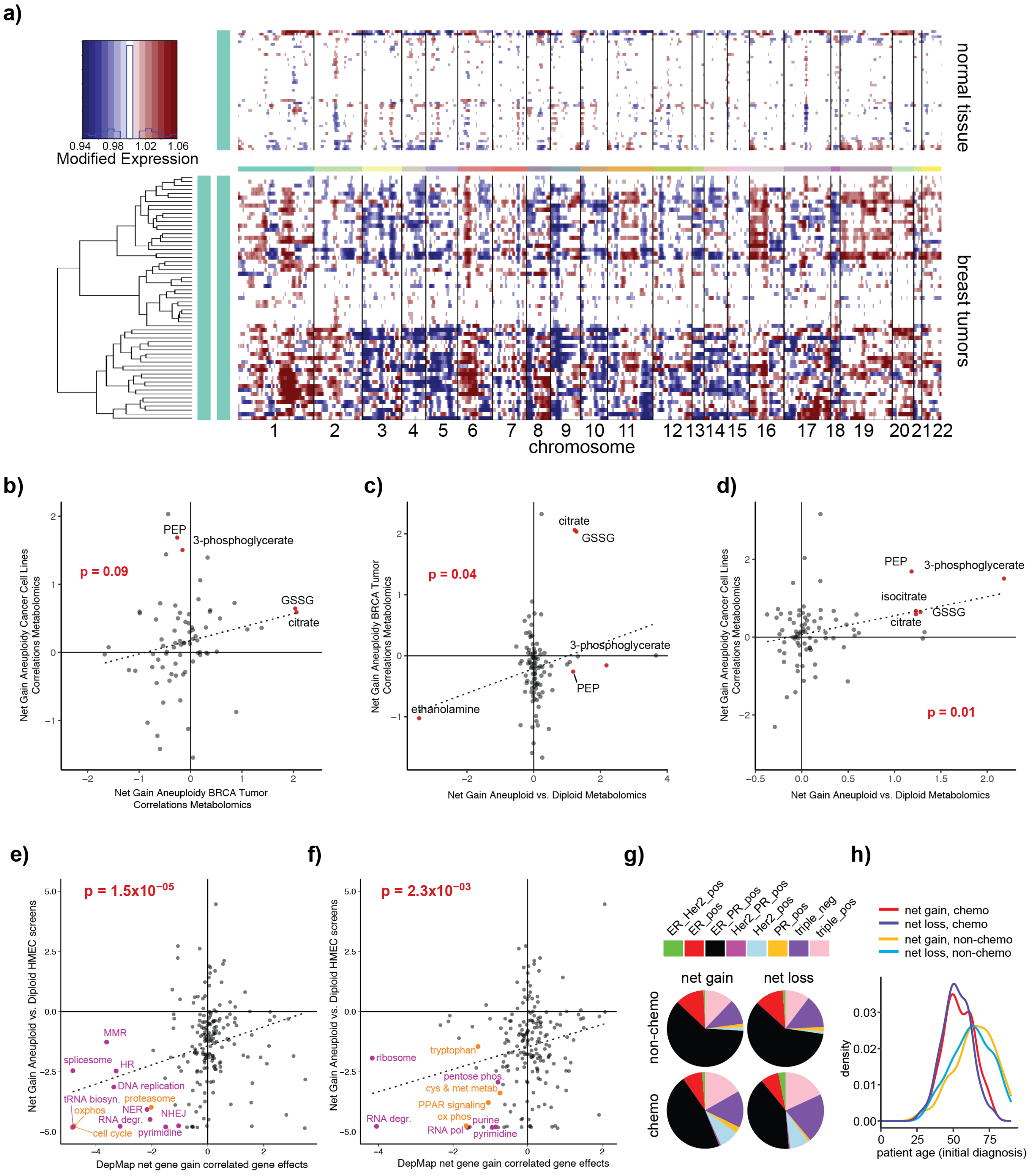

SUPPLEMENTAL FIGURE 6: Net-gain aneuploidy is associated with metabolic phenotypes and is prognostic for response to DNA-damaging or anti-nucleotide chemotherapy in cancer.

a, InferCNV[88] output of breast cancer tumor sample RNA-seq dataset used to infer copy number status for metabolomic phenotype analysis. Samples used for the control group were derived from normal tissue. **b-d** Comparison between the metabolic profile associated with net-gain aneuploidy in this study (HMECs) and in a breast cancer metabolomics dataset (corresponding to the tumors in **(a)**) **(b)**, or in cancer cell line metabolomics datasets **(c)**, or between the tumor and cancer cell line datasets **(d)**. P-values are derived from linear regression analysis. Positive values indicate metabolites that are increased with net-gain aneuploidy; negative values indicate metabolites that are decreased with net-gain aneuploidy. **e, f,** Correlation between the pathway-level epistasis profile obtained from our HMEC net-gain aneuploidy CRISPR screens (y-axis) and the profile obtained from DepMap-based analysis of net-gain aneuploid pan-cancer cell lines (x-axis) **(e)** or just breast cancer cell lines (x-axis) **(f)**. Gene set enrichment analysis using KEGG gene sets of net-gain aneuploidy association rankings in both screens provided the scores for each pathway, which are calculated as the directional - $\log_{10}$(P-value) associated with pathway enrichment or depletion. Negative values indicate pathways that are synthetic-lethal with net-gain aneuploidy. The plotted P-value is derived from linear regression of the two profiles. **g,** Estrogen receptor (ER), Progesterone receptor (PR), and Her2 status (and combinations thereof) distributions among TCGA patients utilized for survival analysis in **Fig. 6g** show no significant difference between net gain and net aneuploidy loss tumor classification groups in the chemotherapy-treated (chemo) and non-chemotherapy-treated (non-chemo) cohorts. See Methods section for description of DNA-damaging/anti-nucleotide/anti-metabolite drug classes that form the "chemo" group in this study. **h,** Distributions of patient age at initial diagnosis within the groups/cohorts used in the survival analysis in **Fig. 6g.** There is no significant difference in age distribution between net gain and net aneuploidy loss tumor classification groups within each treatment cohort, though the chemo-treated cohort has a younger age distribution than the non-chemo cohort.

SUPPLEMENTAL TABLE LEGENDS:

SUPPLEMENTAL TABLE 1: CRISPR library guide sequences.
All sgRNA sequences targeting human protein-coding genes in the CRISPR library.

SUPPLEMENTAL TABLE 2: Essential and enriched gene lists for aneuploid and diploid HMEC CRISPR screens.
Lists of genes that dropped out or were enriched in aneuploid, diploid, or both screens.

SUPPLEMENTAL TABLE 3: Gene-level dropout and enrichment data in a meta-analysis of all aneuploid and diploid CRISPR screens.
Statistical data for each gene in the CRISPR library comparing PD 6 to PD 0 timepoints in the aneuploid and diploid screens.

SUPPLEMENTAL TABLE 4: Meta-analysis comparing PD 6 endpoints of all aneuploid vs. diploid CRISPR screens.
Statistical analysis for each gene in the CRISPR library comparing all aneuploid PD 6 timepoints to all diploid PD 6 timepoints.

SUPPLEMENTAL TABLE 5: Individual aneuploid screen analyses comparing PD 6 endpoints to diploid CRISPR screen PD 6 endpoints.
Statistical analysis for each gene in the CRISPR library comparing PD 6 timepoints of each aneuploid screen to diploid PD 6 timepoints from the same screen batch.

SUPPLEMENTAL TABLE 6: Metabolomics analysis.
Metabolomics analysis for all detected metabolites comparing Brequinar treatment or Brequinar + uridine combination treatment to control conditions in aneuploid and diploid HMECs.

SUPPLEMENTAL TABLE 7: Brequinar transcriptomic response.

RNA-seq analysis for all detected genes comparing Brequinar treatment or Brequinar + UMP combination treatment to control conditions in aneuploid and diploid HMECs.

SUPPLEMENTAL TABLE 8: Gene-level analysis in DepMap cancer cell line data related to net-gain chromosomal imbalance.

Gene effect correlation analysis across cancer cell lines in the DepMap CRISPR dataset with respect to net-gain chromosomal imbalance in cancer cell line genomes.
